# Preventive effects of *Lentinus edodes* on homocysteinemia in mice

**DOI:** 10.3892/etm.2013.1130

**Published:** 2013-05-24

**Authors:** HYUN YANG, INHO HWANG, SUN KIM, CHANGHWAN AHN, EUI-JU HONG, EUI-BAE JEUNG

**Affiliations:** Laboratory of Veterinary Biochemistry and Molecular Biology, College of Veterinary Medicine, Chungbuk National University, Cheongju, Chungbuk 361-763, Republic of Korea

**Keywords:** *Lentinus edodes*, hyperhomocysteinemia, folate, vitamin B12, eritadenine

## Abstract

Homocysteinemia is associated with cardiovascular and neuronal degenerative diseases. Deficiencies of the B vitamins lead to high homocysteine serum levels. *Lentinus edodes* (*L. edodes)* is also known as the Shiitake mushroom and may have beneficial effects on vascular and lipid metabolic diseases, including hypertension, homocysteinemia and lipidemia. In this study, we induced a homocysteinemia-like condition in mice by the administration of a folate- and vitamin B12-deficient diet and evaluated the effect of *L. edodes* on the homocysteinemia-like condition. Homocysteinemia was induced by the administration of a diet deficient in folate and vitamin B12 (DFV) for 6 weeks to mice aged 4–10 weeks. The homocysteinemic mice were treated with *L. edodes* flour (5, 10 and 20%), eritadenine (10 mg/kg) or DFV only (negative control) for 2 weeks. The DFV induced a significant increase in serum homocysteine levels. The increased homocysteine serum levels were reduced by eritadenine and *L. edodes* flour (5, 10 and 20%). Hepatic levels of S-adenosyl-L-homocysteine hydrolase (SAH) were significantly higher under DFV administration and the elevated SAH levels were reduced by treatment with *L. edodes* in a dose-dependent manner. The mRNA expression levels of DNA methyl transferases, DNMT1 and DNMT3a, were reduced in the DFV group, and the reduced levels of DNMT1 and DNMT3a mRNA expression were recovered in the eritadenine and *L. edodes* (5, 10 and 20%) groups. These results suggest that components of *L. edodes*, including eritadenine may have beneficial effects on hyperhomocysteinemia and its therapeutic effects may be involved in the regulation of DNA methylation-related genes in mice.

## Introduction

The Shiitake mushroom, or *Lentinus edodes (L. edodes*) has been recognized for having beneficial bioactivities. Shiitake mushrooms are an important ingredient in Asian foods, since they possess a desirable taste and odor. Additionally, this fungus has an excellent nutritional value with high levels of vitamins B and D 1). A bioactive compounds from *L. edodes*, eritadenine, has been shown to exert anti-hypercholemic effects in previous studies (2,3). In addition to this, eritadenine may affect lipid metabolism through the inhibition of S-adenosyl homocysteine hydrolase (SAH) (4–6).

Homocysteine is a non-protein amino acid that is biosynthesized through the metabolism of methionine (6). Elevated levels of homocysteine have been associated with number of diseases, including heart failure and bone disorders. High homocysteine concentrations increase the susceptibility to endothelial injury, which leads to inflammation in various tissues and may result in ischemic injury and metabolic imbalances (7–9). In humans, normal plasma levels of total homocysteine are 5–15 mmol/l. An increase in this amino acid to 5 *μ*mol/l is associated with an elevated risk of coronary heart disease by 60–80% for males and females (10). In hyperhomocysteinemic patients, high levels of homocysteine are significantly reduced by folic acid and vitamin B12 supplementation (11). Deficiencies in B vitamins (vitamin B6, 9 and 12) may lead to hyperhomocysteinemia (12). In addition, folate and vitamin B12 deficiencies cause DNA damage, which may result from hypomethylation (3,13).

DNA methylation is an important epigenetic mechanism that selectively regulates the expression of targeted genes and is associated with various cardiac diseases (14). Methylation at the promoter region controls gene transcription and in turn attracts histone deacetylases (HDACs). DNA methylation is mediated by DNA methyl transferase (DNMT); this enzyme uses S-adenosyl methionine (SAM) as a methyl group source. DNMTs include DNMT1, 2 and 3. Homocysteine selectively reduces the activity of DNMT1, resulting in increased levels of SAH in human endothelial cells (15).

The nutritional role of dietary *L. edodes* has not yet been elucidated in the mouse model of hyperhomocysteinemia. In the present study, we investigated the effect of *L. edodes* in a mouse model of hyperhomocysteinemia induced by a folate- and vitamin B12-deficient diet (DFV) during the growth stage of the animals (between 4 and 12 weeks of age). The serum and hepatic levels of homocysteine were measured using serum chemistry and high performance liquid chromatography (HPLC). Expression of DNMTs in the liver was also evaluated in order to determine the potential anti-hyperhomocysteinemic effects of *L. edodes*.

## Materials and methods

### Experimental animals

ICR mice (4 weeks-old) were obtained from Koatech (Pyeongtaek, Gyeonggi, South Korea). All animals were housed in polycarbonate cages and acclimated in an environmentally controlled room (temperature, 23±2°C; relative humidity, 50±10%; frequent ventilation; and a 12/12-h light-dark cycle) prior to use. The mice (n=60) were divided into six groups (n=10 per group). Homocysteinemia was induced in five groups by the administration of DFV for 6 weeks.

To assess the preventative effect of *L. edodes* on hyperhomocysteinemia, mice received DFV alone as a negative control (NC), DFV + eritadenine (10 mg/kg) as a positive control (PC) or DFV + 5% (T1; w/w), 10% (T2; w/w) or 20% (T3; w/w) *L. edodes,* from 10- to 12 weeks of age. A sham group received AIN-93M pellets. The body weights of the mice were measured before and after the experiment. The Ethics Committee of Chungbuk National University (Cheongju, Korea) approved all experimental procedures.

### Measurement of SAM, SAH and serum homocysteine levels

Frozen mouse liver samples were homogenized with 0.4 M HClO_4_ buffer. The tissue homogenates were centrifuged at 2,000 × g at 4°C for 20 min. Supernatants were filtered with a 0.45-*μ*m filter. SAM and SAH concentrations were measured with Shimadzu LC-10 HPLC apparatus (Tokyo, Japan) equipped with a 250×4.6-mm Ultrasphere 5-*μ*m ODS Betasil analytical column (Thermo Hypersil-Keystone, Runcorn, UK) according to a previously described protocol (16). Blood was collected from each mouse, transferred to serum separator tubes and centrifuged at 400 × g for 20 min. The supernatant was transferred into 1.5 ml tubes and analysis of serum homocysteine levels was performed using a chemiluminescent immunoassay method with an ADVIA Centaur Assay system (Siemens Medical Solution Diagnostics, Dublin, Ireland) according to the manufacturer’s instructions.

### RNA extraction and quantitative PCR

Total RNA was extracted from mouse liver using TRIzol reagent (Invitrogen Life Technologies, Carlsbad, CA, USA) according to the manufacturer’s instructions. The RNA concentration was determined using an Epoch micro-plate spectrophotometer (BioTek Instruments, Inc., Winooski, VT, USA) at an absorbance of 260 nm and RNA quality was evaluated by electrophoresis in 1% agarose gels. Total RNA (1 mg) was reverse transcribed into first-strand complementary DNA (cDNA) using Moloney murine leukemia virus reverse transcriptase (Invitrogen Life Technologies) and a random primer (9-mer; Takara Bio Inc., Shiga, Japan). Each cDNA sample (1 ml) was amplified by 10 ml 2X SYBR^®^ Premix Ex Taq™ (Takara Bio Inc.) and 10 pmol each primer. Amplification was performed in a 7300 Real time PCR System (Applied Biosystems, Foster City, CA, USA) using the following parameters: denaturation at 95°C for 5 min, then 40 cycles of denaturation at 95°C for 30 sec, annealing at 60°C for 30 sec and extension at 72°C for 45 sec. The oligonucleotide primer sequences used in this study were as follows: 5′-AAC CAA GCA AGA AGT GAA GCC C-3′ (sense) and 5′-GCA AAA TGA GAT GTG ATG GTG G-3′ (antisense) for DNMT1 (product size, 185 bp); 5′-GGA GGA ATG TGC CAA AAC TG-3′ (sense) and 5′-GCA GTT GTT GTT TCC GCA C-3′ (antisense) to amplify DNMT3a (product size, 132 bp); and 5′-AAC AGC ATC GGC AGG AAC-3′ (sense) and 5′-ATC TTT CCC CAC ACG AGG-3′ (antisense) for DNMT3b (product size, 249 bp). The oligonucleotide primer sequences used to amplify β-actin (product size, 131 bp) were: 5′-GGC ACC CAG CAC AAT GAA G-3′ (sense) and 5′-GCA AAA TGA GAT TGT ATG GTG G-3′ (antisense). The relative expression levels of DNMT1 (normalized to the level of β-actin) in each sample were determined using RQ software (Applied Biosystems). All real-time PCR experiments were repeated twice.

### Data analysis

Data are presented as the mean ± standard error of the mean (SEM) and were analyzed with a one-way analysis of variance (ANOVA) followed by Tukey’s multiple comparison test. Statistical analyses were performed using Prism Graph Pad (v.4.0; GraphPad Software Inc., San Diego, CA, USA). P<0.05 was considered to indicate a statistically significant difference.

## Results

### Body weight and daily dietary intake

Differences in body-weight and daily intake between mice who ate a normal diet and those who received DFV for 6 weeks were evaluated. The mice from the six groups had similar body weight gains as shown in Table I. The body weights of mice in the sham, NC and PC (eritadenine) groups did not change in response to the DFV. The groups treated with *L. edodes* flour (10 and 20%) had lower final body weights compared with those of the other groups at 6 weeks; however, the differences in weight were not significant.

### Serum levels of homocysteine in hyperhomocysteinemic mice

**Figure 1.**
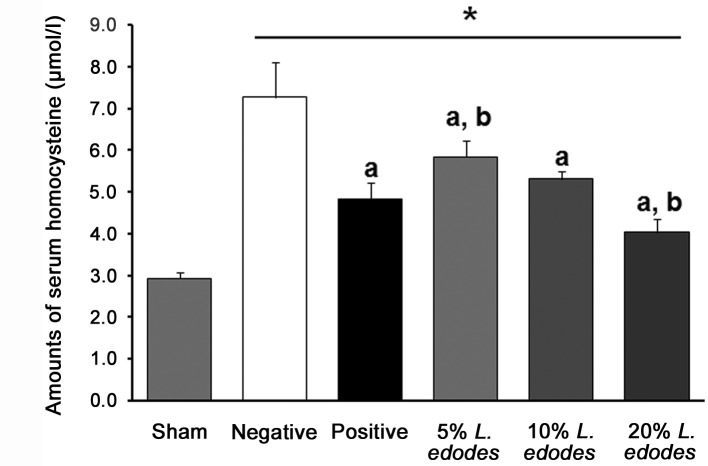
Serum levels of homocysteine with folate- and vitamin B12 deficiency (DFV)-induced hyperhomocysteinemia in mice. The serum homocysteine levels were measured in the six groups: AIN-93M diet only(sham), DFV (negative control), DFV + eritadenine (positive control), DFV + 5% *Lentinus edodes* (T1), DFV + 10% *Lentinus edodes* (T2) and DFV + 20% *Lentinus edodes* (T3). The level of homocysteine was examined by a chemiluminescent immunoassay method (CLIA). ^*^P<0.05 vs. the sham group; ^a^P<0.05 vs. the negative control; ^b^P<0.05 vs. the positive control.

Dietary supplementation with *L. edodes* affected the serum levels of homocysteineine in mice with hyperhomocysteinemia, caused by the DFV (Fig. 1). The DFV resulted in a marked increase in homocysteine levels from 2.9±0.1 to 7.3±0.8 mmol/l after 6 weeks. The addition of dietary eritadenine (10 mg/kg) or *L. edodes* flour (5, 10 or 20%) attenuated the rise in serum homocysteine levels caused by the DFV. In the groups receiving *L. edodes* (5, 10 and 20%), the levels of homocysteine were reduced in a dose-dependent manner compared with those in the NC group.

### Hepatic SAH and SAM levels, and SAM/SAH ratios in hyperhomocysteinemic mice

HPLC was used to measure SAM and SAH levels, and the SAM/SAH ratios in liver tissues were calculated. Hepatic SAH levels were increased by the DFV as shown in Table II. The level of SAH was increased in the NC group. The increase in SAH levels was significantly reduced by eritadenine, as well as by *L. edodes* (5, 10 and 20%) in a dose-dependent manner. In addition, the SAM/SAH ratio was reduced by the DFV and increased by dietary supplementation with eritadenine and *L. edodes* flour (5, 10 and 20%).

### Expression of DNMT (1 and 3a) mRNA in the livers of hyperhomocysteinemic mice

**Figure 2.**
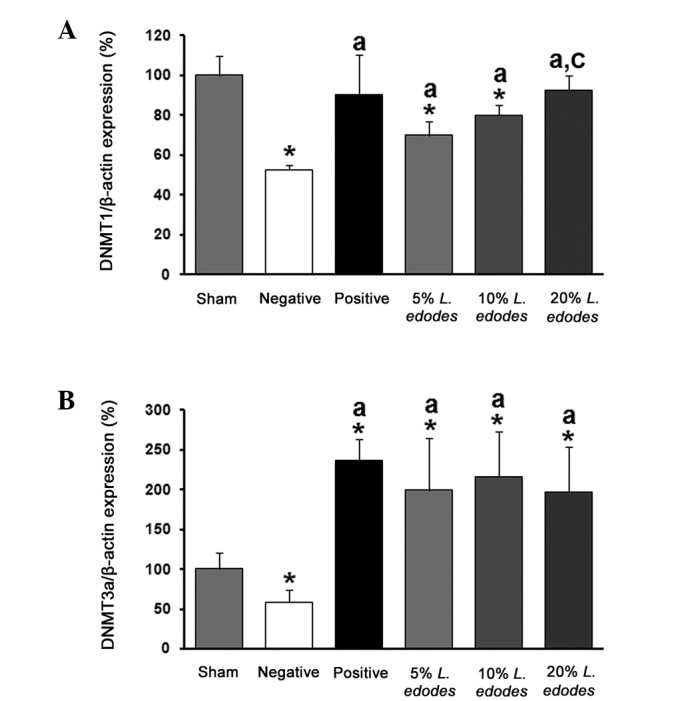
Pattern of DNMT1 and DNMT3a mRNA expression in mice with folate and vitamin B12 deficiency (DFV)-induced hyperhomocysteinemia. Quantitative PCR was performed to detect DNMT1 and DNMT3a mRNA in the livers of mice. Liver tissues were divided into six groups: AIN-93M diet only (sham), DFV (negative control), DFV + eritadenine (positive control), DFV + 5% *Lentinus edodes* (T1), FVD + 10% *Lentinus edodes* (T2) and DFV + 20% *Lentinus edodes* (T3). ^*^P<0.05 vs. the sham group; ^a^P<0.05 vs. the negative control; ^c^P<0.05 vs. 5% *L. edodes*. DNMT, DNA methyl transferase.

To measure the expression of hepatic DNMT1, the mice were separated into six groups (sham, NC, PC, T1, T2, and T3). As shown in Fig. 2, the level of DNMT1 mRNA was reduced by the DFV in the NC group compared with that in the sham group, and was significantly increased by eritadenine supplementation in the PC group. Hepatic DNMT1 mRNA expression was also dose-dependently increased by supplementation with *L. edodes* (5, 10 and 20%). Hepatic DNMT3b mRNA was not abundant in the liver of this mouse model while DNMT3a expression was reduced by DFV compared with the level in the sham group. This inhibitory effect was eliminated by eritadenine and all of doses of *L. edodes* flour (5, 10 and 20%).

## Discussion

*L. edodes* contains considerable concentrations of vitamins (D, B6, B9 and B12) and other beneficial compounds, including eritadenine and dietary fiber (1). This fungus is considered to be useful for treating hypercholesterolemia, inflammation, hypertension and osteoporosis (17–19). In a previous study, we examined the anti-osteoporotic effects of *L. edodes* and the ability of this fungus to induce the expression of duodenal and renal calcium transport channels in mice with osteoporosis-like symptoms (19). In the present study, we investigated the anti-hyperhomocysteinemic effects of *L. edodes* and determined whether it prevents increases in homocysteine levels in mice with dietary folate and vitamin B12 deficiencies. After the mice had received specialized diets for 6 weeks, starting when the animals were 6 weeks old, we observed that dietary *L. edodes* supplementation reduced the serum and hepatic levels of homocysteine in the hyperhomocysteinemic mice. Eritadenine was also shown to have a similar effect.

Dietary supplementation with eritadenine has been reported to effectively suppress guanidine acetic acid (GAA)-induced hyperhomocystenemia in rats (6). However, the mechanism underlying this effect has not been fully elucidated. Analyses of eritadenine content in *L. edodes* identified that the concentration of this compound ranged between 3.2 and 6.3 mg/g dried mushroom (20). The total amount of aministered eritadenine in the PC group (10 mg/kg) was lower compared with the amount contained in the dietary *L. edodes* (>25 mg/kg); however, the anti-hyperhomocysteinemic effects were similar.

The expression of DNMT1 mRNA, which the DFV down-regulated, was increased by eritadenine and dose-dependently increased by *L. edodes* (5, 10 and 20%) in the mice with hyperhomocysteinemia compared with the level in the NC group. Similarly, the level of DNMT3a mRNA was decreased by the DFV; this inhibitory effect was abolished and the level of DNMT3a was elevated by eritadenine and all doses *L. edodes* (5, 10 and 20%) compared with the sham group. A previous study demonstrated that homocysteine selectively reduces the activity of DNMT1 and increases SAH levels in human endothelial cells, but does not affect DNMT3 (15). In another study, DNMT1 and DNMT3 expression was reported to be elevated by homocysteine in human monocytes (21). The levels of DNMT1 and DNMT3 have been demonstrated to be mediated or selectively regulated by homocysteine (15,21). DNMT3 levels have been found to differ between mouse liver, HUVECs and human monocytes, and may vary according to tissue type and species (15,21,22). These results indicate that homocysteine is affected by upregulation of DNA methylation through the suppression of HDAC activity and increased DNMT1 and DNMT3 activities.

In conclusion, the present study demonstrated that supplementation with eritadenine and *L. edodes* (5, 10 and 20%) significantly inhibited the effects of DFV-induced hyperhomocysteinemia in mice. Reduced serum and hepatic homocysteine levels illustrated the beneficial effects of *L. edodes* on hyperhomocysteinemia-like symptoms. Serum and hepatic homocysteine levels were significantly reduced by the administration of eritadenine and *L. edodes* to hyperhomocysteinemic mice with dietary folate and vitamin B12 deficiencies compared with their levels in the NC group. In addition, we examined the expression of DNMT1 and DNMT3a in the livers of mice and demonstrated that hepatic DNMT1 and DNMT3a levels were increased to the levels observed in sham animals by eritadenine and *L. edodes*. Based on our findings, we propose that *L. edodes* may improve hyperhomocysteinemic symptoms due to the beneficial compounds it contains.

## Figures and Tables

**Table I.** Body weight and daily intake.

Group	Weight (g)		Daily intake (g)
	Baseline	8 weeks later	
Sham	37.1±0.9	40.1±0.3	4.2±0.5
Negative	37.2±0.9	41.3±3.4	4.6±0.9
Positive	37.6±0.7	41.0±0.5	4.6±0.6
5% *L. edodes*	37.5±0.9	41.9±0.7	4.2±0.5
10% *L. edodes*	37.8±0.1	39.7±1.3	4.4±0.8
20% *L. edodes*	37.6±0.8	39.1±2.1	4.6±1.0

**Table II.** Hepatic SAH and SAM levels, and SAM/SAH ratios in hyperhomocysteinemic mice.

Group	SAH (nmol/g tissue)	SAM (nmol/g tissue)	SAM/SAH ratio
Sham	5.2±0.2	10.8±0.6	2.1±0.1
Negative	13.5±0.5	7.7±0.4	0.58±0.04
Positive	6.6±0.4^a^	10.3±0.9^a^	1.59±0.19^a^
5% *L. edodes*	8.7±0.4^a^	7.8±0.8	0.90±0.08^a^
10% *L. edodes*	7.5±0.4^a^^b^	8.0±0.8	1.01±0.07^a^^b^
20% *L. edodes*	6.5±0.3^a^^c^	8.2±1.0	1.16±0.08^a^^c^

a P<0.05 vs. negative control;

b P<0.05 vs. 5% *L. edodes*;

c P<0.05 vs. 10% *L. edodes*. SAH, S-adenosyl homocysteine hydrolase; SAM, S-adenosyl methionine.
